# Coronary artery calcium and cystatin C for risk stratification of MACCEs and all‐cause death in symptomatic patients

**DOI:** 10.1002/clc.23959

**Published:** 2022-12-09

**Authors:** Fan Luo, Jun‐Yi Luo, Fen Liu, Ya‐Jing Qiu, Xin‐Xin Tian, Lu Zeng, Zhuo‐Ran Zhang, Xiao‐Mei Li, Yi‐Ning Yang

**Affiliations:** ^1^ Department of Cardiology The First Affiliated Hospital of Xinjiang Medical University Urumqi Xinjiang China; ^2^ Xinjiang Key Laboratory of Cardiovascular Disease Research, Prevention and Treatment of High Incidence Diseases in Central Asia, Clinical Medical Research Institute The First Affiliated Hospital of Xinjiang Medical University Urumqi Xinjiang China; ^3^ Department of Cardiology People's Hospital of Xinjiang Uygur Autonomous Region Urumqi Xinjiang China

**Keywords:** all‐cause death, coronary artery calcium score, cystatin C, major adverse cardiac and cerebrovascular events, risk stratification, symptomatic patient

## Abstract

**Objectives:**

The aim of this study was to examine the independent and joint associations of baseline coronary artery calcium score (CACS) and cystatin C (Cys‐C) with the risk of major adverse cardiac and cerebrovascular events (MACCEs) and all‐cause death in symptomatic populations.

**Methods:**

The study included 7140 patients with symptom of chest pain who underwent cardiac computerized tomography examinations to measure CACS. All of them had serum Cys‐C results. Endpoints were set for MACCEs and all‐cause death events.

**Results:**

A total of 7140 participants were followed for a median of 1106 days. A total of 305 patients had experienced MACCEs and 191 patients had experienced all‐cause death. CACS ≥ 100 and Cys‐C ≥ 0.995 mg/L were independently associated with an increased risk of MACCEs (adjusted hazard ratio [HR]: 1.46; 95% confidence interval [CI]: 1.15–1.85; *p* = .002 and adjusted HR: 1.57; 95% CI: 1.24–2.00; *p* < .001, respectively). Compared with CACS < 100 and Cys‐C < 0.995 mg/L patients, CACS ≥ 100 and Cys‐C ≥ 0.995 mg/L patients had the highest risk of MACCEs and all‐cause death (adjusted HR: 2.33; 95% CI: 1.64–3.29; *p* < .001 and adjusted HR: 2.85; 95% CI: 1.79–4.55; *p* < .001, respectively). Even in patients with CACS < 100, Cys‐C ≥ 0.995 mg/L was also associated with a higher risk of MACCEs and all‐cause death than Cys‐C < 0.995 mg/L (adjusted HR: 1.76; *p* = .003 and adjusted HR: 2.02; *p* = .007, respectively).

**Conclusions:**

The combined stratification of CACS and Cys‐C showed an incremental risk of MACCEs and all‐cause death, reflecting complementary prognostic value. Our results support the combination of the two indicators for risk stratification and event prediction.

## 1 INTRODUCTION

Cardiovascular disease is the main cause of disease burden globally, leading to premature mortality and rising health care costs.^1^, ^2^ The prevalent cases of cardiovascular disease increased from 271 million in 1990 to 523 million in 2019.^3^ About 19 million people died of cardiovascular disease in 2020.^4^ Therefore, risk stratification and predictability of cardiovascular disease are significant for the subsequent management decisions of patients. Considering the significant impact of either over‐ or underestimation of cardiovascular disease risk on lifelong management decisions and health costs, a more accurate risk assessment method was needed to identify not only those at high risk but also those at low risk for appropriate allocation of finite resources in a cost‐effective approach.

Coronary artery calcium (CAC) is a highly specific marker of coronary atherosclerosis, quantified using the Agatston method to yield the coronary artery calcium score (CACS).^5^ CACS has been proved to be a powerful independent predictor of major adverse cardiovascular events and all‐cause death.^6^ Compared with traditional cardiovascular disease risk factors, CACS is recommended as a more accurate risk stratification and event prediction method. CACS can reclassify risks based on traditional risk factors to identify individuals with higher or lower risks.^7^, ^8^ However, The CACS based on the Agatston method also has certain limitations. Plaques with low calcium density may be associated with large lipid nuclei, predisposing to cardiovascular events, while plaques with high calcium density may be more stable.^9^, ^10^, ^11^ In addition, the Agatston method cannot assess the number of calcified coronary arteries and the degree of concentration and dispersion of coronary calcification,^12^ which has been questioned.

Cystatin C (Cys‐C) is a cysteine protease inhibitor with a low molecular weight produced at a constant rate by all nucleated cells. As a sensitive marker of renal function, Cys‐C has been proved to be a more accurate assessment of renal function than creatinine, especially in patients with mild to moderate renal insufficiency, and its serum concentration is not affected by age, sex, or muscle mass.^13^ Past studies have found that elevated Cys‐C levels were significantly and independently associated with an increased risk of cardiovascular death and all‐cause death among older adults in the community, patients with chronic kidney disease, and patients with acute heart failure.^14^, ^15^, ^16^ However, few studies have used Cys‐C for risk stratification and event prediction in patients with symptom of chest pain.

In people with the symptom of chest pain suggestive of coronary heart disease (CHD), it is uncertain whether the CACS and Cys‐C can identify people at different risks of cardiovascular events or death, and whether their combination can provide complementary prognostic information for further risk stratification to identify people with higher or lower risks. To address this issue, we evaluated the association of baseline CACS and Cys‐C with future major adverse cardiac and cerebrovascular events (MACCEs) and all‐cause death in symptomatic patients.

## 2 METHODS

### 2.1 Patients

We consecutively included 9226 patients with symptom of chest pain suggestive of CHD between December 2013 and April 2020 in the First Affiliated Hospital of Xinjiang Medical University. All of these patients were referred for cardiac computed tomography (CT) by their cardiologists. Exclusion criteria included patients who had noncardiac chest pain (*n* = 23), a history of coronary revascularization (percutaneous coronary intervention [PCI], *n* = 814), coronary artery bypass graft surgery (CABG) (*n* = 238), history of myocardial infarction (MI) (*n* = 145) or missing results of Cys‐C (*n* = 295). In total, 7711 patients were enrolled. The study was approved by the ethics committee of the First Affiliated Hospital of Xinjiang Medical University (K202106‐02).

### 2.2 CAC scoring

All the patients underwent cardiac CT for quantification of CAC. Cardiac CT was performed using multi‐detector row CT scanners consisting of 64‐row or greater. Calcium was defined as a focus of at least 3 contiguous pixels with a CT density >130 Hounsfield units. The total calcium burden of the coronary arteries was quantified by the scoring algorithm proposed by Agatston et al.^5^ Patients were classified into two groups: those with CACS < 100 and those with CACS ≥ 100.^17^

### 2.3 Cystatin C

Blood samples were obtained from all subjects after overnight fasts of ≥8 h to evaluate blood biochemistry. Serum Cys‐C was measured by the immunoturbidimetry method (N Latex Cystatin, Beckman Coulter Chemistry Analyzer AU5800 Serie). The serum Cys‐C of patients were divided into two groups according to the cut‐off point of MACCEs’ receiver operating characteristic (ROC).

### 2.4 Outcomes

MACCEs were the primary composite outcomes. MACCEs were defined prospectively from baseline examination as time to first instance of coronary revascularization (PCI and CABG), MI, fatal and nonfatal stroke, malignant arrhythmias, refractory heart failure, or cardiovascular death. Cardiovascular death was defined as death due to MI, malignant arrhythmias, refractory heart failure, or cardiogenic shock. The secondary endpoint comprised all‐cause death. The deadline for follow‐up is April 2021. MACCEs and all‐cause death information were obtained from patient telephone interviews, contact with the patient's physicians, and hospital records. If the patients could not be contacted successfully by us through their direct phone number three times in 1 week, or if the patient could not provide accurate information, that patient was tabulated as lost to follow‐up.

### 2.5 Statistical analysis

The optimum cut off value for Cys‐C was calculated for the detection of a high risk of MACCEs using ROC curve analysis. The best cut off point for Cys‐C was assessed by the Youden index (J), which represented the maximum sum of sensitivity and specificity. Participants were evaluated based on the risk stratification of CACS and Cys‐C (low risk: CACS < 100 or Cys‐C < 0.995 mg/L. High risk: CACS ≥ 100 or Cys‐C ≥ 0.995 mg/L). The baseline characteristics were represented as median and quartiles (Q1–Q3) for continuous variables and percentages for categorical variables, and were compared by Mann–Whitney or Kruskal–Wallis test and Chi‐square analysis, respectively. Concordance between CACS and Cys‐C hazard stratification was assessed using Cohen's kappa coefficient. Cumulative incidence of MACCEs and all‐cause death were estimated by the Kaplan‐Meier. Cox proportional hazards regression was used to evaluate the associations of CACS and Cys‐C with MACCEs and all‐cause death. The relationship between CACS and Cys‐C with MACCEs and all‐cause death risk were assessed using smoothing splines. The area under the ROC curve was to evaluate the prognostic discriminatory capacity for predicting MACCEs and all‐cause death. All statistical analyses were performed using SPSS for windows version 26.0 and R version 4.1.2. A *p* < .05 was considered statistically significant.

## 3 RESULTS

### 3.1 Baseline characteristics

In all, 571 (7.4%) patients were missed, and the remaining 7140 patients who had follow‐up data of incident MACCEs and all‐cause death were finally analyzed. The median age of the patients was 63 years, and 64.9% were male. The cohort was followed up for a median of 1106 days (quartile 1 = 622 days, quartile 3 = 1787 days). CACS was low‐risk stratification (CACS < 100) in 3777 (52.9%) patients (male 52.5%, female 53.6%). Cys‐C was low‐risk stratification (Cys‐C < 0.995 mg/L) in 4460 (62.5%) patients, including 62.5% of male and 62.4% of female. Concordance of hazard stratification between CACS and Cys‐C demonstrated an agreement rate of 54.6% (Cohen's K: 0.079; 95% confidence interval [CI]: 0.055–0.103). Similar results were observed for male (Cohen's K: 0.078; 95% CI: 0.051–0.105) and female (Cohen's K: 0.082; 95% CI: 0.043–0.121) (Table S1).

Baseline characteristics of the 7140 symptomatic patients are shown in Table 1. As compared with patients with CACS ≥ 100, patients with CACS < 100 were younger and less likely to have diabetes or hypertension. As compared with patients with Cys‐C ≥ 0.995 mg/L, patients with Cys‐C < 0.995 mg/L were younger, but had higher rates of diabetes. In the combined stratification of Cys‐C and CACS, patients with CACS ≥ 100 and Cys‐C ≥ 0.995 mg/L were older, and more likely to have diabetes or hypertension than those with CACS < 100 and Cys‐C < 0.995 mg/L.

**Table 1:** Baseline characteristic of study participants stratified by CACS, Cys‐C, and a combination of both

	Total (*N* = 7140)	CACS			Cys‐C		
		<100 (*n* = 3777)	≥100 (*n* = 3363)	*p* value	<0.995 mg/L (*n* = 4460)	≥0.995 mg/L (*n* = 2680)	*p* value
Age, years	63 (55–71)	60 (53–68)	66 (58–74)	<.001	61 (53–68)	67 (59–74)	<.001
Male	4635 (64.9)	2435 (64.5)	2200 (65.4)	.402	2897 (65.0)	1738 (64.9)	.929
Never smoked	4821 (67.5)	2578 (68.3)	2243 (66.7)	.160	2976 (66.7)	1845 (68.8)	.064
BMI, kg/m^2^	26.3 (25.3–26.8)	26.3 (25.4–26.8)	26.3 (25.2–26.8)	.332	26.3 (24.9–27.0)	26.3 (26.0–26.3)	.028
Diabetes	2237 (31.3)	1053 (27.9)	1184 (35.2)	<.001	1437 (32.2)	800 (29.9)	.037
Hypertension	4958 (69.4)	2485 (65.8)	2473 (73.5)	<.001	3082 (69.1)	1876 (70.0)	.426
Total cholesterol, mmol/L	4.1 (3.4–4.8)	4.2 (3.6–4.9)	4.0 (3.3–4.7)	<.001	4.2 (3.5–4.9)	4.0 (3.3–4.7)	<.001
LDL cholesterol, mmol/L	2.8 (2.2–3.2)	2.8 (2.3–3.3)	2.8 (2.1–3.2)	<.001	2.8 (2.2–3.3)	2.8 (2.3–3.0)	.003
HDL cholesterol, mmol/L	1.1 (1.0–1.3)	1.1 (1.0–1.3)	1.1 (0.9–1.3)	.206	1.1 (1.0–1.3)	1.1 (1.0–1.2)	<.001
Triglycerides, mmol/L	1.6 (1.1–2.4)	1.7 (1.1–2.5)	1.5 (1.1–2.4)	<.001	1.7 (1.2–2.5)	1.5 (1.1–2.3)	<.001
CACS	87 (20–295)	23 (6–52)	318 (174–666)	<.001	75 (18–252)	110 (27–361)	<.001

CACS and Cys‐C				
Cys‐C <0.995 mg/L and CACS <100 (*n* = 2499, 35%)	Cys‐C <0.995 mg/L and CACS ≥100 (*n* = 1961, 27.5%)	Cys‐C ≥0.995 mg/L and CACS <100 (*n* = 1278, 17.9%)	Cys‐C ≥0.995 mg/L and CACS ≥100 (*n* = 1402, 19.6%)	*p* value
58 (52–65)	63 (56–71)	63 (55–71)	70 (62–76)	<.001
1611 (64.5)	1286 (65.6)	824 (64.5)	914 (65.2)	.86
1682 (67.3)	1294 (66)	896 (70.1)	949 (67.7)	.108
26.3 (25.0–26.9)	26.3 (24.8–27.1)	26.3 (26.1–26.4)	26.3 (25.9–26.3)	.090
732 (29.3)	705 (36.0)	321 (25.1)	479 (34.2)	<.001
1639 (65.6)	1443 (73.6)	846 (66.2)	1030 (73.5)	<.001
4.3 (3.6–5.0)	4.1 (3.4–4.8)	4.1 (3.4–4.7)	4.0 (3.3–4.6)	<.001
2.8 (2.3–3.3)	2.8 (2.0–3.3)	2.8 (2.3–3.1)	2.8 (2.2–3.0)	<.001
1.1 (1.0–1.3)	1.1 (0.9–1.3)	1.1 (1.0–1.2)	1.1 (0.9–1.2)	.003
1.7 (1.2–2.6)	1.6 (1.1–2.4)	1.6 (1.1–2.4)	1.5 (1.1–2.2)	<.001
**—**	**—**	**—**	**—**	**—**

*Note*: Values are median (interquartile range) or *n* (%).

Abbreviations: BMI, body mass index; CACS, coronary artery calcium score; Cys‐C, cystatin C; HDL, high‐density lipoprotein; LDL, low‐density lipoprotein.

### 3.2 Association of CACS with MACCES and all‐cause death

A total of 305 MACCEs and 191 all‐cause death events were observed during follow‐up. Higher incidence rates of MACCEs and all‐cause death were observed in CACS ≥ 100 group (16.4 events/1000 person‐years [PY] and 10.6 events/1000 PY, respectively) compared with CACS < 100 group (9.5 events/1000 PY and 5.4 events/1000 PY, respectively) (Table S2). Cumulative incidence curves also showed that the group with higher levels of CACS had a higher subsequent incidence of MACCEs and all‐cause death (Figure S1). Similar results were observed for male and female. In our continuous analysis, higher levels of CACS were associated with progressively increased risk of MACCEs and all‐cause death (Figure S2A,B). In categorical analyses, elevated CACS level (CACS ≥ 100 vs. CACS < 100) was still independently associated with an increased risk of MACCEs after adjusting for traditional cardiovascular risk factors (adjusted hazard ratio [HR]: 1.46; 95% CI: 1.15–1.85; *p* = .002) (Table 2). Similar results were observed for male (adjusted HR: 1.56; 95% CI: 1.17–2.07; *p* = .002), but not in female (adjusted HR: 1.24; 95% CI: 0.79–1.92; *p* = .350). However, elevated CACS level (CACS ≥ 100 vs. CACS < 100) only had a nominal association with the risk of all‐cause death (crude HR: 1.95; 95% CI: 1.46–2.62; *p* < .001; adjusted HR: 1.31; 95% CI: 0.97–1.78; *p* = .077) (Table 2). Similar results were observed for male (adjusted HR: 1.36; 95% CI: 0.94–1.98; *p* = .103) and female (adjusted HR: 1.20; 95% CI: 0.71–2.02; *p* = .500).

**Table 2:** Independent and joint association of elevated CACS and Cys‐C with the risk of MACCEs and all‐cause death

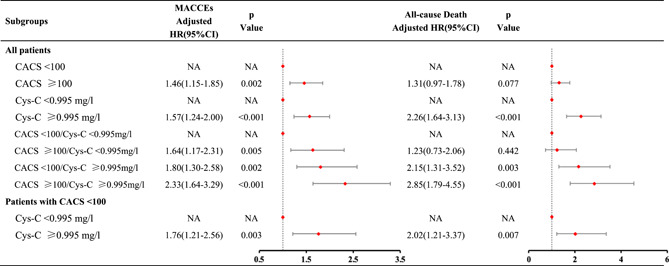

*Note*: Cox proportional hazards regression models adjusted for age, sex, smoking status, body mass index, diabetes, hypertension, triglycerides, total cholesterol, low‐density lipoprotein cholesterol, and high‐density lipoprotein cholesterol.

Abbreviations: CI, confidence interval; HR, hazard ratio; MACCE, major adverse cardiac and cerebrovascular events; other abbreviations as in Table 1.

### 3.3 Association of CYS‐C with MACCES and all‐cause death

In our analysis, higher incidence rates of MACCEs and all‐cause death were observed in Cys‐C ≥ 0.995 mg/L group (17.6 events/1000 PY and 13.8 events/1000 PY, respectively) compared with Cys‐C < 0.995 mg/L group (9.5 events/1000 PY and 4.0 events/1000 PY, respectively) (Table S2). Cumulative incidence curves also showed that the group with higher levels of Cys‐C had a higher subsequent incidence of MACCEs and all‐cause death (Figure S3). Similar results were observed for male and female. In our continuous analysis, higher levels of Cys‐C were associated with progressively increased risk of MACCEs and all‐cause death (Figure S2C,D). In categorical analyses, elevated Cys‐C level (Cys‐C ≥ 0.995 mg/L vs. Cys‐C < 0.995 mg/L) was still independently associated with an increased risk of MACCEs after adjusting for traditional cardiovascular risk factors (adjusted HR: 1.57; 95% CI: 1.24–2.00; *p* < .001) (Table 2). Similar results were observed for male (adjusted HR: 1.62; 95% CI: 1.22–2.16; *p* = .001), but weakened in female (adjusted HR: 1.49; 95% CI: 0.96–2.33; *p* = .076). Elevated Cys‐C level (Cys‐C ≥ 0.995 mg/L vs. Cys‐C < 0.995 mg/L) was also independently associated with an increased risk of all‐cause death after adjusting for traditional cardiovascular risk factors (adjusted HR: 2.26; 95% CI: 1.64–3.13; *p* < .001) (Table 2). Similar results were observed for male (adjusted HR: 2.07; 95% CI: 1.39–3.10; *p* < .001) and female (adjusted HR: 2.58; 95% CI: 1.49–4.49; *p* = .001).

### 3.4 Joint association of CAC score and CYS‐C with MACCE and all‐cause death

**Figure 1:**
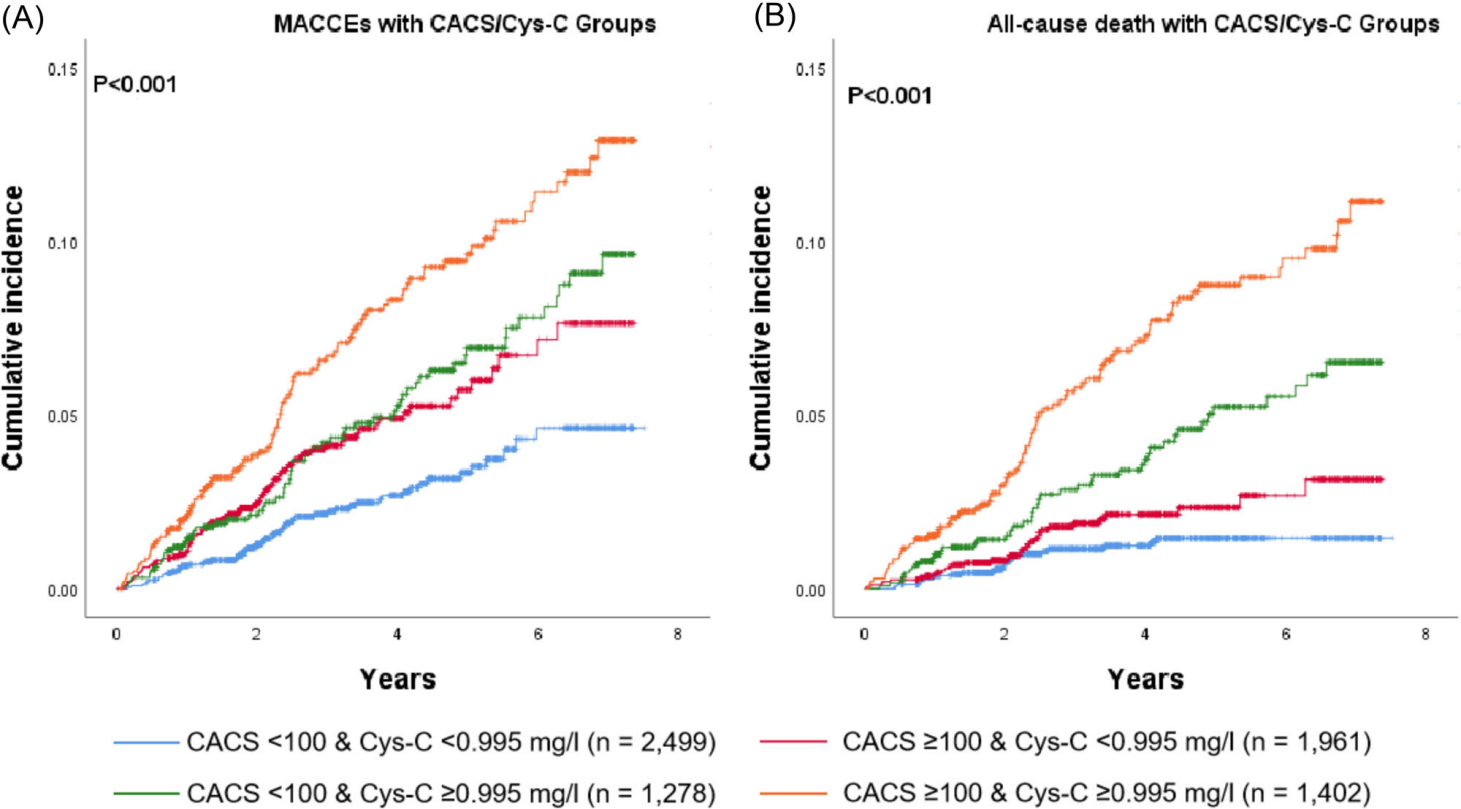
Cumulative MACCEs and all‐cause death events incidence across CACS and Cys‐C groups. (A) Cumulative MACCEs incidence across CACS and Cys‐C groups. (B) Cumulative all‐cause death events incidence across CACS and Cys‐C groups. The Kaplan–Meier illustrates the highest MACCEs and all‐cause death incidence in the CACS ≥ 100 with Cys‐C ≥ 0.995 mg/L group. CACS, coronary artery calcium score; MACCE, major adverse cardiac and cerebrovascular event

MACCEs and all‐cause death outcomes stratified by CACS and Cys‐C were shown in Figure 1, Table S2. Among our patients stratified by CACS and Cys‐C thresholds (100 and 0.995 mg/L), the highest MACCEs and all‐cause death incidences were observed in the CACS ≥ 100 and Cys‐C ≥ 0.995 mg/L group (20.8 events/1000 PY and 17.4 events/1000 PY, respectively), while the lowest incidences were observed in the CACS < 100 and Cys‐C < 0.995 mg/L group (7.0 events/1000 PY and 3.1 events/1000 PY, respectively). Similar results were observed for male and female, but the MACCEs incidence of Cys‐C ≥ 0.995 mg/L/CACS ≥ 100 in female did not increase significantly compared with Cys‐C ≥ 0.995 mg/L/CACS < 100 (13.7 vs. 13.4 events/1000 PY). In our study, for higher levels of CACS and Cys‐C, the risk of MACCEs and all‐cause death increased (Table 2). Compared with patients in the Cys‐C < 0.995 mg/L/CACS < 100 group, the multivariable adjusted HR (95% Cl) of MACCEs was 1.64 (1.17–2.31) for patients with Cys‐C < 0.995 mg/L/CACS ≥ 100, 1.80 (1.30–2.58) for Cys‐C ≥ 0.995 mg/L/CACS < 100, and 2.33 (1.64–3.29) for patients with Cys‐C ≥ 0.995 mg/L/CACS ≥ 100. The corresponding values of all‐cause death were 1.23 (0.73–2.06), 2.15 (1.31–3.52), and 2.85 (1.79–4.55) respectively. Notably, as binary variables, both CACS and Cys‐C were independent predictors of MACCEs when CACS, Cys‐C, and traditional risk factors were analyzed together in the cox model. Similar results of all‐cause death were observed for Cys‐C but not for CACS.

**Figure 2:**
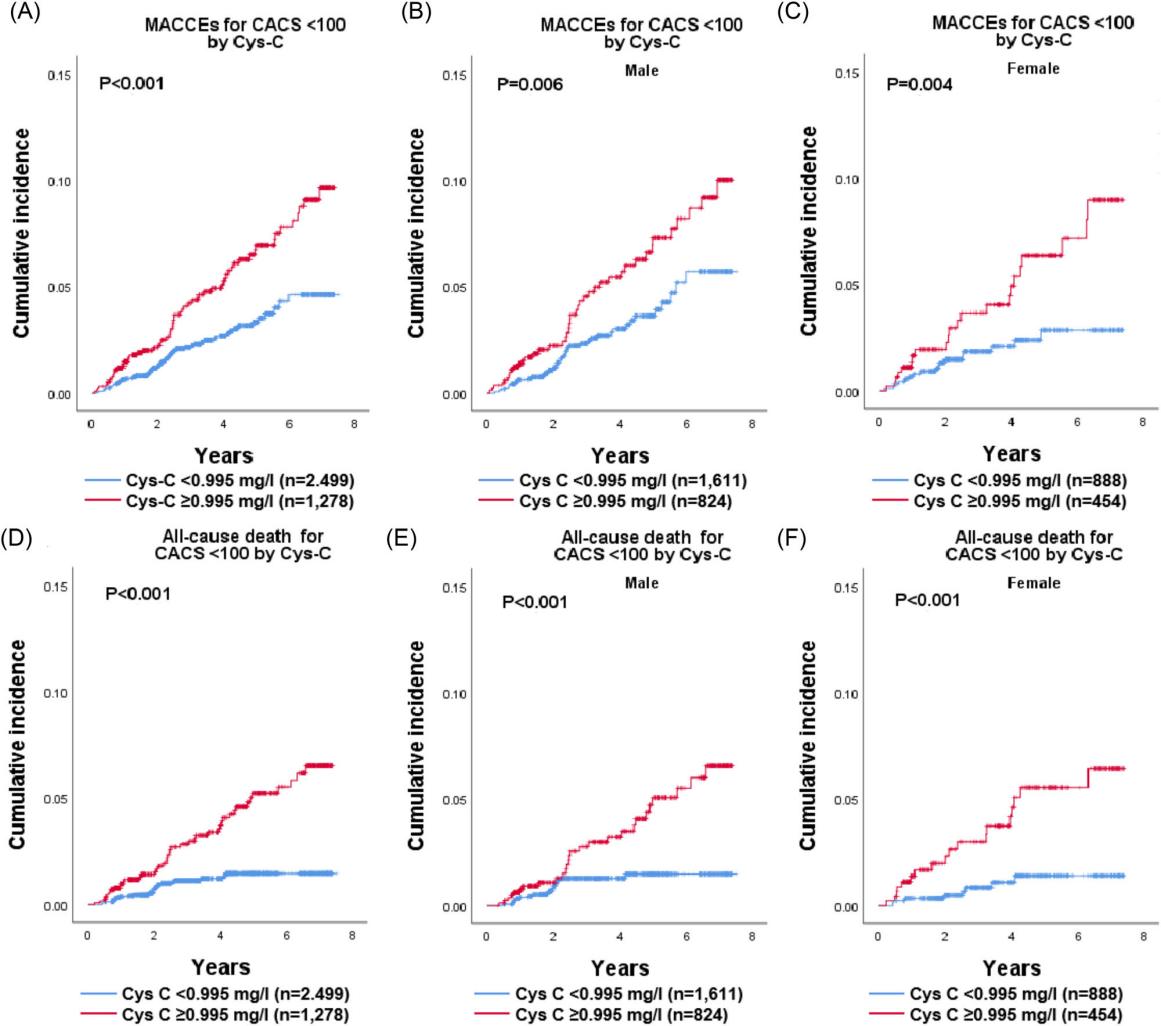
Cumulative MACCEs and all‐cause death events incidence by Cys‐C groups in patients with CAC < 100. Kaplan–Meier plots the cumulative incidence of MACCEs and all‐cause death events by Cys‐C levels (<0.995 mg/L, blue line; ≥0.995 mg/L, red line) in patients with CAC < 100 (A and D, respectively), male with CAC < 100 (B and E, respectively), and female with CAC < 100 (C and F, respectively). Even among patients with CAC < 100, patients with Cys‐C ≥ 0.995 mg/L had a higher cumulative incidence of MACCEs and all‐cause death events than those with Cys‐C < 0.995 mg/L. Similar results were observed for male and female. CACS, coronary artery calcium score; MACCE, major adverse cardiac and cerebrovascular event

Patients with Cys‐C < 0.995 mg/L (62.5% of participants) had a similar risk of MACCEs and all‐cause death as those with CACS < 100 (52.9% of participants) (9.5 vs. 9.5 events/1000 PY and 4.0 vs. 5.4 events/1000 PY, respectively) (Table S2). Similar findings were observed for male and female. A total of 123 MACCEs and 71 all‐cause death events occurred among the patients of CAC < 100. Patients with higher level of Cys‐C (≥0.995 mg/L) showed an increased risk of MACCEs in patients with CACS < 100 (14.1 vs. 7.0 events/1000 PY; adjusted HR: 1.76; 95% CI: 1.21–2.56; *p* = .003) (Figure 2A–C, Table 2, Table S2). Similar findings were observed for male (14.5 vs. 7.8 events/1000 PY; adjusted HR: 1.59; 95% CI: 1.01–2.51; *p* = .047) and female (13.4 vs. 5.5 events/1000 PY; adjusted HR: 2.15; 95% CI: 1.09–4.24; *p* = .027). Patients with higher level of Cys‐C (≥0.995 mg/L) also showed an increased risk of all‐cause death in patients with CACS < 100 (9.8 vs. 3.1 events/1000 PY; adjusted HR: 2.02; 95% CI: 1.21–3.37; *p* = .007) (Figure 2D–F, Table 2, Table S2). Similar incidence rates were observed for male (9.2 vs. 3.3 events/1000 PY) and female (10.8 vs. 2.6 events/1000 PY). Adjusted analyses showed a significant association between Cys‐C ≥ 0.995 mg/L and all‐cause death in female (adjusted HR: 2.81; 95% CI: 1.15–6.84; *p* = .023) with CAC < 100, but not in male (adjusted HR: 1.67; 95% CI: 0.88–3.16; *p* = .118).

Among patients with completed follow‐up (*N* = 7140), the area under the ROC curve (AUC) of CACS for incidence MACCEs/all‐cause death were 0.59 and 0.63, respectively. The AUC of Cys‐C for incidence MACCEs/all‐cause death were 0.61 and 0.70, respectively. When CACS and Cys‐C were combined, the AUC for incidence MACCEs/all‐cause death were increased to 0.63 and 0.72, respectively (Figure S4).

## 4 DISCUSSION

### 4.1 Association of CACS with MACCES and all‐cause death

As a marker of subclinical coronary atherosclerosis burden, CAC functions as a summary measure of individual lifetime exposure to coronary risk factors and vascular susceptibility versus resilience to those.^18^ CACS has been described as a strong independent predictor of adverse cardiovascular outcomes and all‐cause death events in the asymptomatic general population and provides accurate risk stratification information.^6^, ^19^, ^20^ Consistent with most previous studies on CACS, in people with symptom, our analysis demonstrated that patients in the high‐risk CACS group were more likely to have subsequent MACCEs and all‐cause death compared with patients in the low‐risk CACS group, and the higher the CACS, the higher the risk of MACCEs and all‐cause death. After adjusting traditional cardiovascular risk factors, consistent with previous studies, the association between CACS and MACCEs remained independent and significant, especially among male patients. Contrary to previous studies, the association between CACS and all‐cause death has weakened, but CACS still showed good predictive value for MACCEs.

The 2018 AHA/ACC/Multi‐Society Cholesterol Guidelines and the 2019 ACC/AHA Primary Prevention Guidelines formally endorsed CACS as a personalized risk management tool for personalized risk stratification,^21^, ^22^ and our study suggests that this ability may also be applicable to symptomatic people. One study found that the cost‐effective of using CAC testing to guide statin therapy was the same as the 2013 ACC/AHA guideline criteria for statin therapy, and cardiac CT may be a more necessary test for symptomatic individuals.^23^ Therefore, using CACS for risk stratification and event prediction to guide treatment decisions may be more cost‐effective for this population.

### 4.2 Association of CYS‐C with MACCES and all‐cause death

Past studies have demonstrated an independent association between Cys‐C levels and cardiovascular death and all‐cause death.^14^, ^15^, ^16^ In community‐based older adults, elevated Cys‐C levels were significantly associated with an increased risk of all‐cause and cardiovascular death, and the association was more potent than creatinine and creatinine‐based glomerular filtration rates.^14^ And Cys‐C also had good prognostic value in patients with chronic kidney disease and heart failure.^15^, ^16^ Our analysis showed that elevated levels of Cys‐C were independently associated with an increased risk of all‐cause death in symptomatic populations. When cardiovascular death in previous studies was extended to include MACCEs, the association with Cys‐C was still observed, independent of traditional cardiovascular risk factors, especially in males.

Cys‐C, as a sensitive marker of renal function, was associated with the risk of MACCEs, which most researchers have accepted. Even mild to moderate decline in renal function was an independent risk factor for cardiovascular death, and renal function also reflects a vascular state such as inflammation, endothelial function, and coagulation function.^24^, ^25^, ^26^ Cys‐C has been shown to be a more accurate assessment of actual renal function than creatinine and creatinine‐based estimated glomerular filtration rate, especially in people with mild to moderate renal decline, and can capture a state of preclinical kidney disease associated with an increased risk of late renal disease and cardiovascular disease.^27^ Therefore, a good correlation between Cys‐C and actual renal function may be the basis for the stratification of cardiovascular disease risk by Cys‐C. In our analysis, we observed a good ability of Cys‐C to stratify the risk of MACCEs and all‐cause deaths. Even in patients with CACS < 100, Cys‐C could further stratify risk and identify those at higher risk. Although a large‐scale Mendelian study has demonstrated no causal relationship between Cys‐C and cardiovascular disease,^28^ suggesting that the use of Cys‐C as a therapeutic target may be insufficient evidence, it cannot be denied that Cys‐C can play an important role in later treatment decision‐making as a tool for risk stratification and event prediction.

### 4.3 Joint association of CAC score and CYS‐C with MACCE and all‐cause death

A novel finding of our study is that the association of these two risk markers with the risk of MACCEs is independent of each other in symptomatic patients. The level of Cys‐C was independently associated with the risk of all‐cause death, while CACS was weakened. The combination has more accurate risk stratification and events predictive value for subsequent cardiovascular events and all‐cause death, especially in male. Concordance analysis shows a poor consistency between CACS and Cys‐C stratification, suggesting that CACS and Cys‐C results could identify different patients with different risk characteristics. The risk of MACCEs and all‐cause death varies with the combination stratification of the two markers, indicating that the prognostic information is complementary. Not only can they not be substituted for each other, but favors their conjoined use.

When the results of the two tests are concordant, patients with Cys‐C ≥ 0.995 mg/L and CACS ≥ 100 have a nearly threefold increase in the incidence of MACCEs and an almost sixfold increase in the all‐cause death rate compared with those with Cys‐C < 0.995 mg/L and CACS < 100. After adjusting traditional risk factors, there is also a 2.3‐fold increase in the risk of MACCEs and a 2.8‐fold increase in the risk of all‐cause death. Conjoined testing allows for identifying 55% of patients as either low or high risk, allowing for more precise risk stratification. Discordant CACS and Cys‐C results also provide complementary prognostic information. Although CACS < 100 is considered to be a low‐medium risk, patients with Cys‐C ≥ 0.995 mg/L are also associated with a nearly twofold increase in the incidence of MACCEs and an almost threefold increase in the all‐cause death compared with those with Cys‐C < 0.995 mg/L, and 1.8‐fold increase in the risk of MACCEs and 2.1‐fold increase in the risk of all‐cause death after adjusting for traditional risk factors. Cys‐C can identify patients at higher or lower risk in patients with CACS < 100, which increase the value of using CACS independent stratification.

The CACS is a recognized, cost‐effective way of stratifying risk that may be more necessary and cost‐effective for symptomatic patients. Cys‐C is a widely used, simple and readily available indicator of renal function. Therefore, We advocate a combination of the two for risk stratification. Because the risk stratification of CACS and Cys‐C have similar event rates, our results support Cys‐C as an independent indicator for risk stratification when CACS is not available.

### 4.4 Study limitations

First, this is a single‐center study with symptomatic patients, limiting our results’ generalizability to the general population.

Second, residual confounding cannot be excluded given the observational nature of the analysis. Data gathered by self‐report is limited by patient recall, and thus subject to recall bias.

Third, the cut points of Cys‐C need to be validated in separate data before they can be used clinically. Several large clinical studies set the cut‐off point for risk stratification of Cys‐C as 1 mg/L,^27^ while the cut‐off value calculated in this paper by the ROC subject curve is 0.995 mg/L, which is close to the above study. It is also necessary to verify a more accurate risk stratification boundary of Cys‐C, which is conducive to the consistency of studies.

Finally, it has been reported that factors other than renal function, such as glucocorticoid use and thyroid function, might influence cystatin C levels.^29^, ^30^ This study does not eliminate the possible influence of these factors.

### 4.5 Clinical Perspectives

**Competency in Medical Knowledge**: In symptomatic patients, CAC score in combination with Cys‐C allows for more accurate risk stratification of MACCEs and all‐cause death to identify individuals at different risks, and the Cys‐C level can further stratify risk in patients with low CAC score (CACS < 100).

**Translational Outlook 1**: Prospective studies are needed to further evaluate the combined ability of CAC score and Cys‐C to stratify risk and guide treatment decisions, and to validate it in different populations.

**Translational Outlook 2**: CAC score in combination with Cys‐C may be of importance for further treatment strategies in symptomatic populations, and when CAC score is not readily available, Cys‐C may be helpful in making further treatment decisions for this population.

## 5 CONCLUSIONS

Among patients with symptoms at baseline, our study found that elevated CACS and Cys‐C levels were independently associated with an increased risk of MACCEs. After adjustment for cardiovascular risk factors, Cys‐C was independently associated with all‐cause death events, while CACS only had a nominal association with the risk of all‐cause death. The combined stratification of the two indicators showed an incremental risk of MACCEs and all‐cause death, reflecting complementary prognostic value, and was used to separate populations at different risks of MACCEs and all‐cause death. Our study extends risk stratification using CACS or Cys‐C alone to a combination of the two and may suggest a new cardio‐renal axis risk stratification approach. The value of this approach for treatment decision‐making needs to be confirmed in future studies.

## CONFLICTS OF INTEREST

The authors declare no conflicts of interest.

## Supporting information

Supporting information.

## Data Availability

Data available on request from the authors. The data that support the findings of this study are available from the corresponding author upon reasonable request.
