# Supplementary material for: Coronary artery calcium and cystatin C for risk stratification of MACCEs and all‐cause death in symptomatic patients
Source: Clin Cardiol. 2022 Dec 9;46(2):195–203. doi: 10.1002/clc.23959 (PMC9933108; doi:10.1002/clc.23959)
Supplement: Supplementary file 1 — Supporting information. [file CLC-46-195-s001.doc]

**Supplemental Figure Legends**

**Supplemental Figure 1. Cumulative MACCEs and All-cause Death Events Incidence by CACS (<100, ≥100) Groups.**

Kaplan Meier plots the cumulative incidence of major adverse cardiac and cerebrovascular events (MACCEs) and all-cause death events by coronary artery calcium score (CACS) levels **(<100, blue line; ≥100, red line)** in the overall cohort **(A and D, respectively)**, male **(B and E, respectively)**, and female **(C and F, respectively)**. Patients with CACS ≥100 had a higher cumulative incidence of MACCEs and all-cause death events than those with CACS <100. Similar results were observed for male and female.

**Supplemental Figure 2. Association Between CACS and Cys-C and the Risk of MACCEs and All-cause Death.**

**(A and B)** The association between CACS and the hazard ratio for MACCEs and all-cause death. **(C and D)** The association between Cystatin C (Cys-C) and the hazard ratio for MACCEs and all-cause death. In continuous analysis, higher levels of CACS and Cys-C were associated with progressively increased risk of MACCEs and all-cause death. Abbreviations as in **Supplemental Figure 1**.

**Supplemental Figure 3. Cumulative MACCEs and All-cause Death Events Incidence by Cys-C (<0.995 mg/l, ≥0.995 mg/l) Groups.**

Kaplan Meier plots the cumulative incidence of MACCEs and all-cause death events by Cys-C levels **(<0.995 mg/l, blue line; ≥0.995 mg/l, red line)** in the overall cohort **(A and D, respectively)**, male **(B and E, respectively)**, and female **(C and F, respectively)**. Patients with Cys-C ≥0.995 mg/l had a higher cumulative incidence of MACCEs and all-cause death events than those with Cys-C <0.995 mg/l. Similar results were observed for male and female. Abbreviations as in **Supplemental** **Figures 1 and 2**.

**Supplemental Figure 4. ROC Curves for MACCEs and All-cause Death.**

**(A)** The receiver-operating characteristic (ROC) curves for MACCEs: CACS only (area under the curve [AUC] 0.59; 95% confidence interval [CI]: 0.56 to 0.62, p <0.001 **[red line]**). Cys-C only (AUC 0.61; 95% Cl: 0.58 to 0.64, p <0.001 **[green line]**). CACS plus Cys-C (AUC 0.63; 95% Cl: 0.60 to 0.66, p <0.001 **[blue line]**).

**(B)** The ROC curves for all-cause death: CACS only (AUC 0.63; CI: 0.59 to 0.67, p <0.001 **[red line]**). Cys-C only (AUC 0.70; 95% Cl: 0.66 to 0.74, p <0.001 **[green line]**). CACS plus Cys-C (AUC 0.72; 95% Cl: 0.68 to 0.75, p <0.001 **[blue line]**). Abbreviations as in **Supplemental** **Figures 1 and 2**.

Supplemental Figure 1


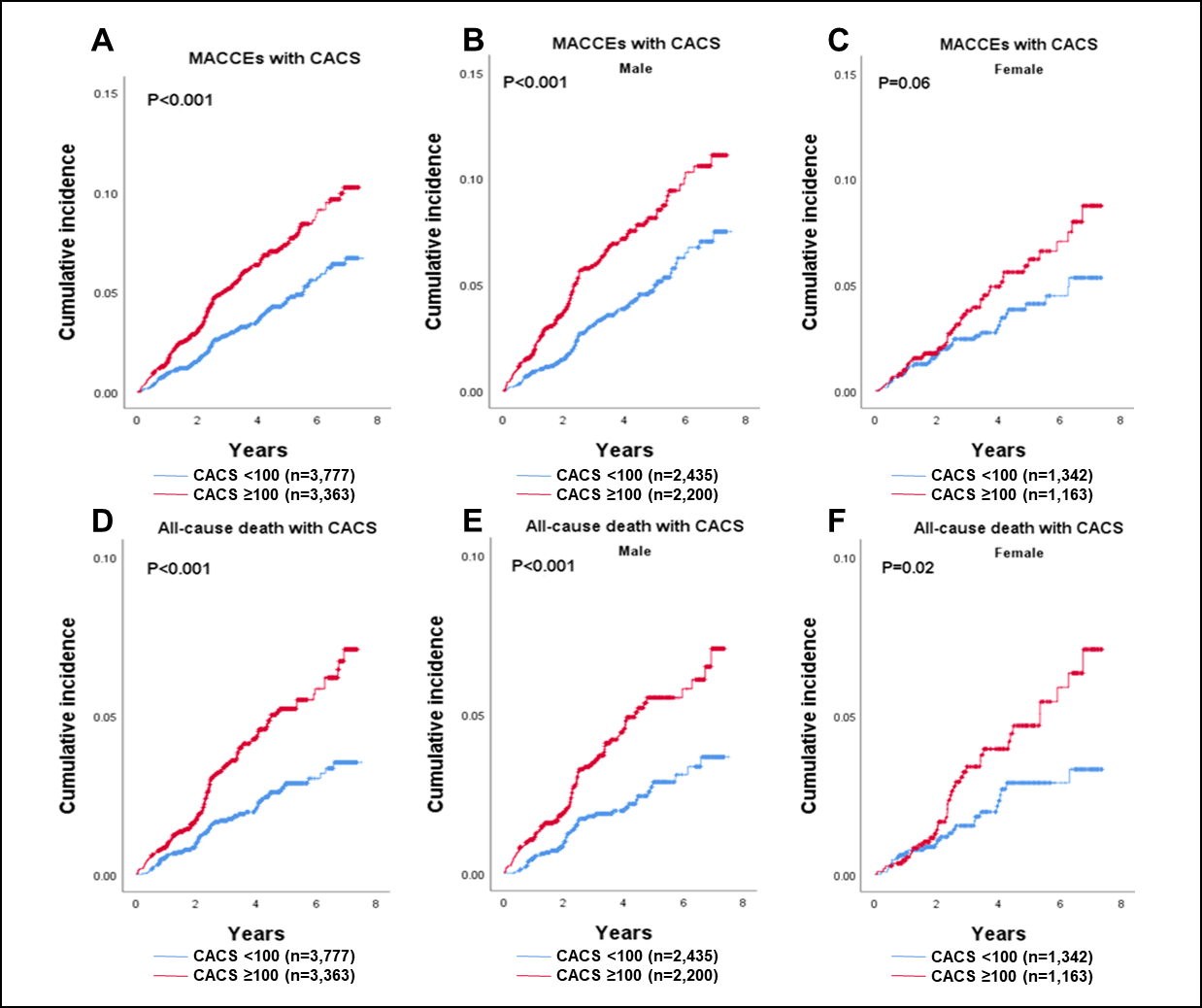


Supplemental Figure 2


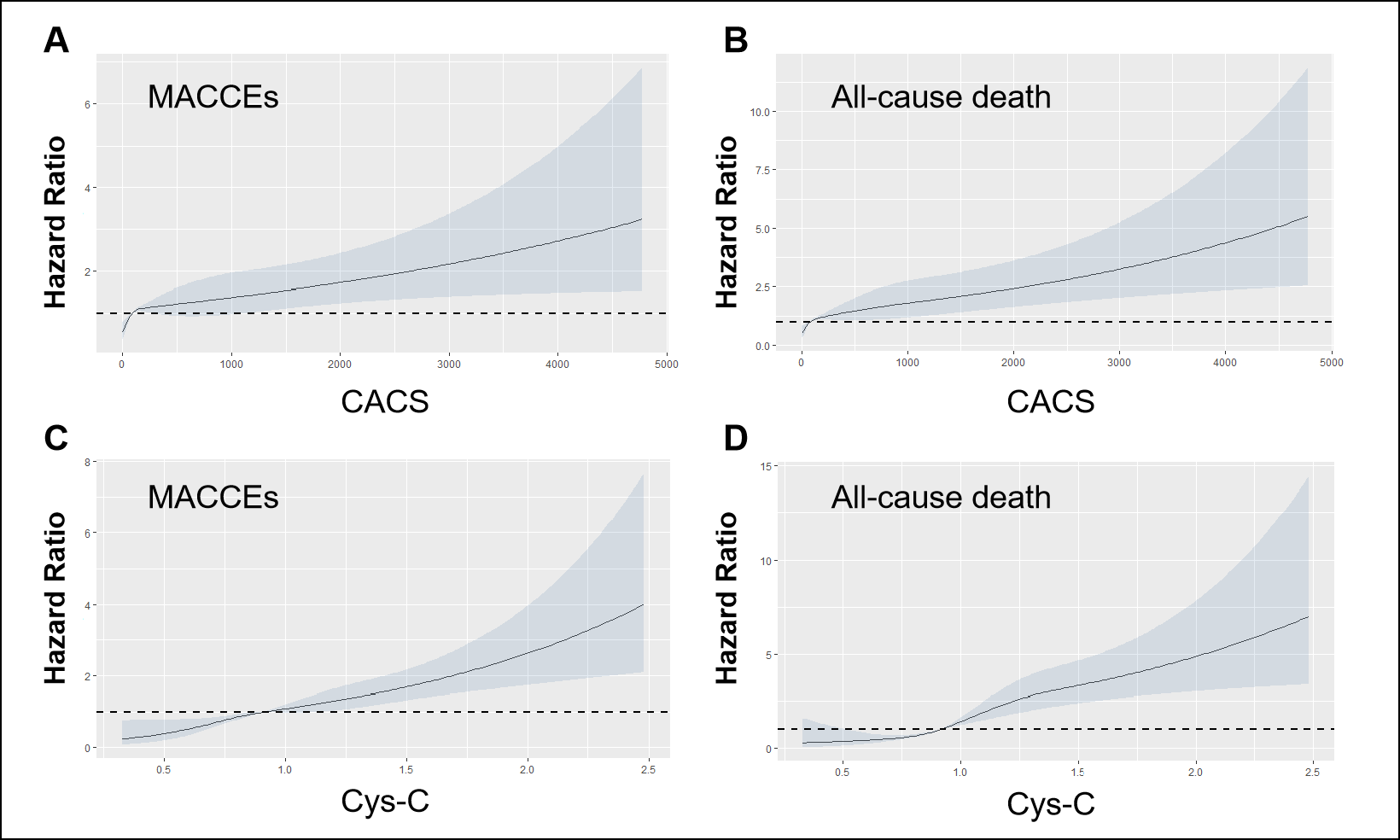


Supplemental Figure 3


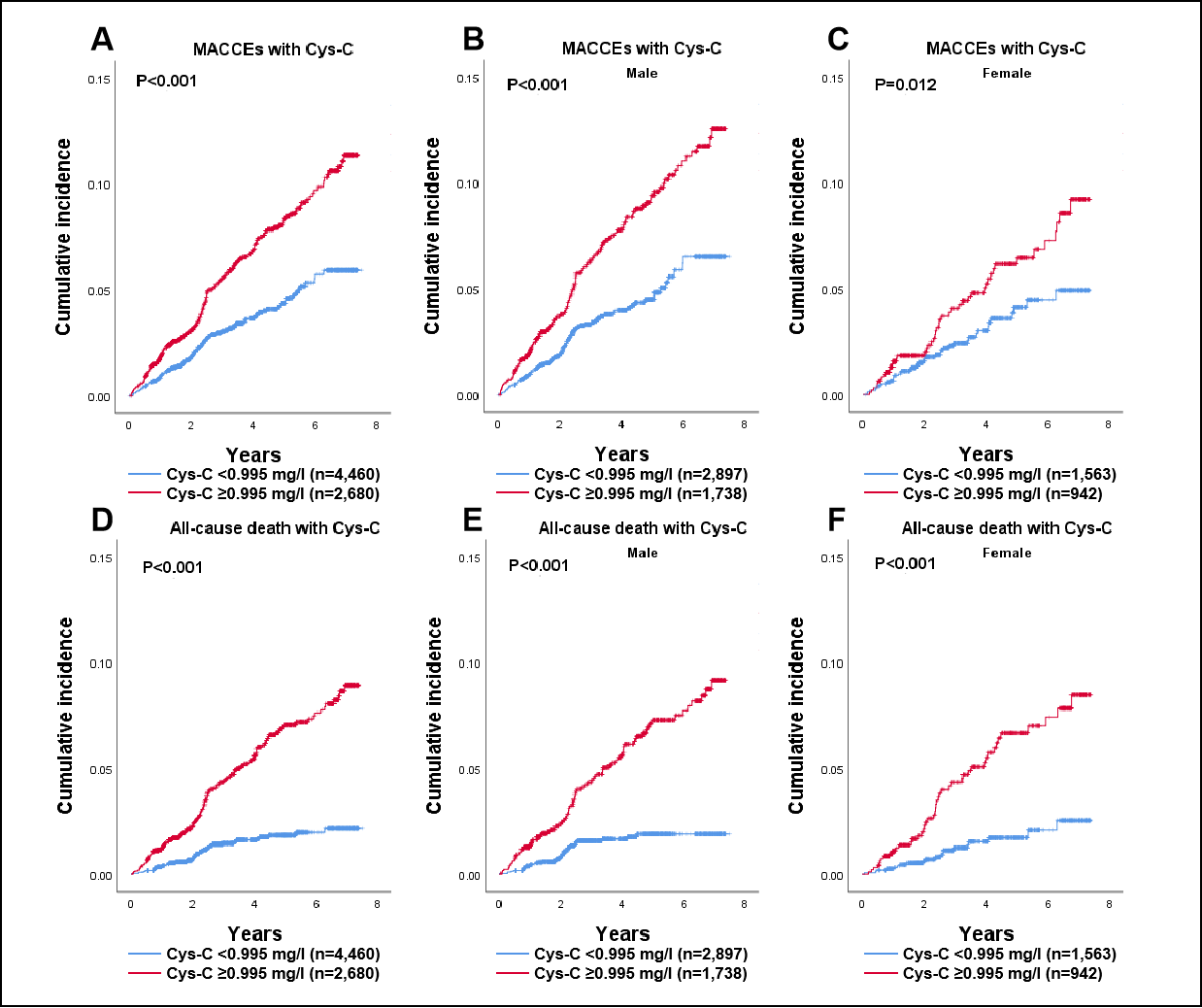


Supplemental Figure 4


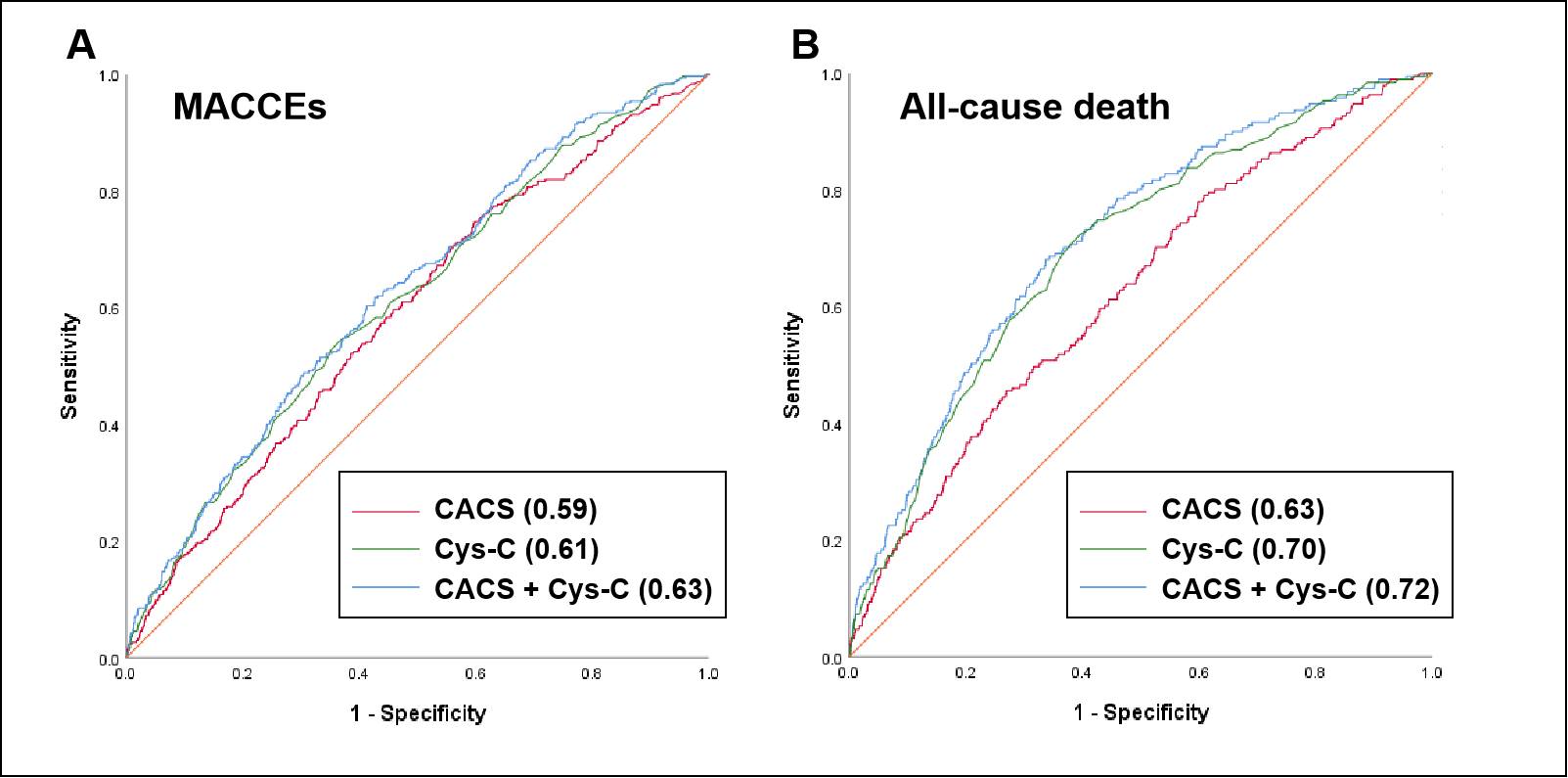


Supplemental Table 1：Concordance analyses between CACS and Cys-C categories for the entire cohort and by sex.

| **Participants** | **CACS and Cys-C concordance** | | | **Agreement** | **Kappa** | **95% CI** |
| --- | --- | --- | --- | --- | --- | --- |
| **Overall** |  | **Cys-C < 0.995mg/l** | **Cys-C ≥ 0.995mg/l** | 0.546 | 0.079 | 0.055-0.103 |
|  | **CACS < 100** | 2499 | 1278 |  |  |  |
|  | **CACS ≥ 100** | 1961 | 1402 |  |  |  |
| **Male** |  | **Cys-C < 0.995mg/l** | **Cys-C ≥ 0.995mg/l** | 0.545 | 0.078 | 0.051-0.105 |
|  | **CACS < 100** | 1611 | 824 |  |  |  |
|  | **CACS ≥ 100** | 1286 | 914 |  |  |  |
| **Female** |  | **Cys-C < 0.995mg/l** | **Cys-C ≥ 0.995mg/l** | 0.549 | 0.082 | 0.043-0.121 |
|  | **CACS < 100** | 888 | 454 |  |  |  |
|  | **CACS ≥ 100** | 675 | 488 |  |  |  |

CACS = coronary artery calcium score; Cys-C = Cystatin C; CI = confidence interval.

Supplemental Table 2. MACCEs and All-cause Death Event Rates Stratified by CACS, Cys-C, and a Combination of Both for the Entire Cohort and by Sex.

|  | **CACS** | |  | **Cys-C** | |  | **Cys-C and CACS** | | | | | |
| --- | --- | --- | --- | --- | --- | --- | --- | --- | --- | --- | --- | --- |
|  | **<100** | **≥100** |  | **<0.995**  **mg/l** | **≥0.995**  **mg/l** |  | **Cys-C <0.995 mg/l /**  **CACS <100** | **Cys-C <0.995 mg/l /**  **CACS ≥100** | | **Cys-C ≥0.995 mg/l /**  **CACS <100** | | **Cys-C ≥0.995 mg/l /**  **CACS ≥100** |
| Overall cohort |  |  |  |  |  |  |  |  | |  |  | |
| Crude MACCEs rate  per 1000 PY | 9.5 | 16.4 |  | 9.5 | 17.6 |  | 7.0 | 12.8 | | 14.1 | 20.8 | |
| Crude All-cause death rate  per 1000 PY | 5.4 | 10.6 |  | 4.0 | 13.8 |  | 3.1 | 5.2 | | 9.8 | 17.4 | |
| Male |  |  |  |  |  |  |  |  | |  |  | |
| Crude MACCEs rate  per 1000 PY | 10.2 | 18.6 |  | 10.3 | 19.8 |  | 7.8 | 13.7 | | 14.5 | 24.8 | |
| Crude All-cause death rate  per 1000 PY | 5.4 | 11.0 |  | 4.1 | 14.1 |  | 3.3 | 5.1 | 9.2 | | 18.6 | |
| Female |  |  |  |  |  |  |  |  |  | |  | |
| Crude MACCEs rate  per 1000 PY | 8.3 | 12.4 |  | 8.0 | 13.6 |  | 5.5 | 11.3 | 13.4 | | 13.7 | |
| Crude All-cause death rate  per 1000 PY | 5.5 | 9.7 |  | 3.8 | 13.1 |  | 2.6 | 5.3 | 10.8 | | 15.3 | |

Values are n unless otherwise indicated.

MACCE = major adverse cardiac and cerebrovascular events; PY = person years. other abbreviations as in **Supplemental Table 1**.
